# Report of a Base Laboratory in Mesopotamia for 1916

**Published:** 1918-10

**Authors:** 


### MEDICINE

***Report of a Base Laboratory in Mesopotamia for 1916, with Special Reference to Water-Borne Diseases*.** By Captain T. K. BONEY, R. A. M. C., and Captain L. G. CROSSMAN, R. A. M. C. and Lieutenant (U. L.) C. L. BOULENGER. *Journal of the Royal Army Medical Corps*, April 1918, Vol. XXX, N° 4, page 409.

The authors give a short account of the results obtained at a British General Hospital laboratory, dealing with the diseases prevalent in Mesopotamia among the troops during 1916, including a few brief remarks as to climate and general considerations which may assist a preliminary understanding of the conditions prevailing there.

The climatic conditions explain the prevalence of malaria and the waterborne diseases among the troops, which diseases show a steady increase from March onward, reaching a maximum in July and August.

*Enteric Group Diseases.* Of 650 cases diagnosed by all methods during the year, only 21 per cent. were found to be true typhoid, while 65 per cent. were due to Bacillus paratyphosus A.

The first case of paratyphoid B was that of an orderly nursing in the paratyphoid ward. Five cases were diagnosed during April, and an increase occurred in May which reached its maximum in June. In fact more than fifty per cent. of all the cases diagnosed occurred during these two months, blood cultures being positive on several occasions. Since then the incidence of the organism has fallen to four or five cases a month, nearly all of which have come from the Euphrates area. Investigation was carried out under three headings:

(a) Isolation of the organism from the blood.
(b) Isolation of the organism from the stools.
(c) Agglutination reactions.

Stool examination was commenced in January and carried on intermittently until the end of April, after which it practically ceased, to be resumed again at the beginning of October. It was chiefly relied upon in those cases seen too late for blood culture — which was made systematically on all possible cases — in which agglutination reactions were likely to be modified by reason of inoculation with T. A. B.

*Blood Cultures.* Identification was based upon:

1. Reaction and amount of gas, if any, in mannite tube.
2. Character of growth on agar and motility.
3. Reaction to specific serum.

The behavior of the paratyphoids in mannite peptone water differed markedly. As a rule paratyphosus B formed acid and gas vigorously — usually to the extent of about one-third of the Durham tube in twenty-four hours.

A certain number of anomalous organisms were isolated from time to time which could not be referred to any group, although they did not appear likely to be contaminations.

In four cases organisms giving all the sugar reactions of the paratyphoid group were met with, but they did not agglutinate, or only very slightly.

At the beginning of October the percentage of positive results sent in from other units was very small compared with those done in this hospital. This may be due to bad selection of cases by medical officers, or may also be accounted for by delay in reaching the laboratory. The figures show that 32.6 per cent. of positive were obtained of all cases, whereas taken separately only 15 per cent. of outside cases yielded positive results as against 34.4 per cent. of cases from this hospital.

*Stools.* Climatic conditions rendered systematic stool plating very difficult, and as a result the method was only followed in a few special cases, reliance being placed on blood culture and Widal tests for the diagnosis of members of the enteric group. Of 108 examinations, B. typhosus was recivered 5 times and B. paratyphosus A eleven times. B. paratyphosus B was never isolated. Direct plating on Mac-Conkey seemed to be the best method for B. paratyphosus B. At the end of twenty-four hours, suspicious colonies were fixed, examined for motility and the sugar reaction tested. The specific serum test was then applied as for organisms isolated from the blood.

*Agglutination Tests* are the least desirable means of search, but hot-weather difficulties and the required length of stay in hospital for stool or urine examinations rendered recourse thereto necessary. Marked agglutination to A or B has been taken as presumptive evidence of infection by the corresponding organism, even if the T-titre was also high. The possibility of mistaking a true T. infection for an A or a B under these conditions would appear to be remote, since it is generally agreed that T. infection produces, as a rule, little group agglutination for A or B, whereas in A or B infections usually the first noticeable change is a rise in the titre to B. typhosus. It is much more necessary, therefore, to be on guard against diagnosing as a T. infection one which is really due to A. or B.; the true nature of the case becoming apparent later on by the appearance of agglutins for these organisms, generally by the end of the third week. Thus subsequent agglutination tests have necessitated the alteration of a certain number of cases. To warrant a diagnosis of true typhoid in a T. inoculated person, a high or rising titre to T. was required, with none to A and little or none to B.

Stool examination, uncertain as a means of detecting the " Carrier ", is even more uncertain as a means of diagnosing large numbers of acute cases during a rush of work. Possibly more energetic hemoculture in all suspected early cases may help in part to solve future difficulties in diagnosis.

*Agglutination Technic.* The strains used were all reliable ones.

two being brought from France and the third from India. Carbolized agar emulsions were made from subcultures, and retained their agglutinability through the hot weather.

In performing the tests the dilutions were made on a glass plate ruled with grease pencil, then drawn up into lengths of glass tubing, of bore about three millimeters, and incubated for eighteen hours at $37^{0}$ C when the reading was made, using a 12 lens.

The total number of agglutination tests made during the year was 1,283, and the number of diagnoses made 520, or 40 per cent. Of these 520, enteric fever accounts for 113 = 21 per cent., paratyphoid A for 326 = 62 per cent. and paratyphoid B for 82 = 16 per cent.

*Inoculation.* Out of figures taken of cases in which the organism was obtained from the blood, — apart from the fact that cases of enteric fever form only 20 per cent. of the total of these, — only 27 per cent. had received inoculation within one and a half years, and 50 per cent. had never been inoculated at all.

With regard to T. A. B., if its value is as great as that of the old typhoid vaccine, it is a little surprising to find the incidence of paratyphosus A so high in men inoculated within six months of the onset. As a matter of fact nearly all these cases occurred within four months of inoculation, and some a good deal less than this.

*Cholera.* Cholera was first noticed among the troops towards the end of April and the cases were isolated after an examination of the stools. From this time on, there was a steady stream of cases during the hot weather. The outbreak died out by the end of September, having never become alarming. A mortality of about 40 per cent. was recorded.

In forming a positive diagnosis, the following points were taken into consideration :

(1) Naked eye appearance of the stools.

(2) Characters of smear stained with dilute carbolfuchsin.

(3) Culture in peptone water and examination of the top layer for vibrios after eight to ten hours' incubation. The use of Witte peptone was found invaluable in this connection, and frequently gave results when other good peptone, although sugar-free, was negative.

(4) Reaction against specific high-titer serum.

(5) Cholera-red reaction. The hemolytic test was not done in any case.

Of the twenty-eight cases in which a vibrio was isolated, twenty-two were diagnosed on the growth of a vibrio in peptone water, which was morphologically V. cholerae, and which was agglutinated in the manner specified. In the remaining six no agglutination occurred.

*The Dysenteries.* Amebic dysentery, whether it is endemic in this country or not, has certainly been responsible for a great deal of sickness during the hot weather, and of even commoner occurrence has been the bacillary form of the disease. Speaking generally of cases with dysenteric symptoms, the maximum of the curve of incidence was reached in August and September, as in the case of enteric group disease, after which it gradually fell again.

It was not possible to examine systematically these cases, but fifty-nine of the stools were plated, all of them containing blood and mucus. B. dysenteriae Flexner was isolated in seven, and B. dysenteriae Shiga in two. Microscopical examination of eighty stools, also containing blood and mucus, showed E. histolytica in eleven.

At the beginning of September it became possible to make a detailed examination of all cases, and 890 cases of intestinal disorder were investigated during the last four months of the year.

These cases fell into two groups:

(1) Cases of acute dysentery.

(2) Cases in which some diarrheal symptoms were present, and in which there may or may not have been a history of a previous acute dysentery.

The method adopted was as follows:

1. Microscopical examination was first made, and the protozoölogical findings noted.

2. Those containing blood and mucus (i. e., group 1., acute dysentery, and not showing the presence of B. Histolytica) were then plated.

3. Stools containing no blood or mucus were not plated.

This was the general procedure, broken only by a few exceptions.

One hundred and eight cases, or 51.7 per cent., of those submitted to bacteriological examination were proved to be due to infection by B. dysenteriae; and the relative incidence of the two forms of the disease will be: amebic 26 per cent. and bacillary 35 per cent. of the total number of cases examined. Two cases of mixed amebic and bacillary infection were noted: namely, (1) E. histolytica and B. Shiga; and (2) E. histolytica and B. Flexner.

There still remains 39 per cent of acute cases in which the causal agent was not determined.

The Medical Advisory Committee made a report in which it was stated that in the Mesopotamian area, as in other war areas, bacillary dysentery is the predominant type, and that had the cases investigated been in the main local admissions instead of transfers from up-river, the proportion of bacterially proved bacillary cases would have been still higher.

*Protozoölogical Findings in Group I* (*Acute dysentery*) *and in Group II* (*Chronic dysentery and diarrheal conditions*). From the month of September, 1916, to the end of that year nearly 1,300 stools were dealt with, representing 890 separate cases, — all British patients. The uniform method of collecting specimens was the use of bed-pans, and smears from the feces were examined in salt solution (warmed to blood temperature when necessary during the winter months) and Weigert's iodine solution, according to the method recommended by Lieutenant-Colonel C. N. Wenyon, R. A. M. C., in his recent publications on the subject. As a result of the examination of these 890 cases, it becomes possible to state that over a quarter (25.9 per cent.) of the acute dysenteries at this hospital, during the last four months of the year, were of amoebic origin.

*Malaria.* In January benign tertian parasites were found in only 11 cases and subtertian in 1, out of a total of 90 examinations. In May an increase was noticeable, when the number of examinations had risen to about 150 with about sixteen per cent. positive. The monthly average was 200 per month with seventeen per cent. positive. The total number of slides examined was between 2,200 and 2,300 during the year.

*Relapsing Fever.* Forty-seven cases were diagnosed by finding spirilla in the blood. Only one case terminated fatally, and the epidemic was arrested and terminated by means of strict isolation and disinfective measures.

*Jaundice.* Blood cultures were done on five cases of jaundice with low pyrexia. All were negative.

*Cerebro Spinal Fever.* The meningococcus was found in the cerebro-spinal fluid of six cases. It could not be cultivated on blood-smeared agar on any occasion, although once or twice it was present in enormous numbers.

Diagnosis was based upon the polymorphonuclear nature of the exudate and the presence of a Gram-negative diplococcus morphologically resembling the meningococcus, a certain number of which were inside the cells.

*Diphtheria.* This disease was not infrequent. Of a total of 22 cases which occurred, the majority were those in men in the same regiment, and occurred during August and September. The method of making an inoculation upon the white of a recently hard-boiled egg, inverted in a urine specimen glass containing a little water to keep it moist, proved to be of considerable practical value, when the heat made difficult the preparation of serum media.

*Vaccines.* Prophylactic vaccines were made for cholera and enteric group. The cholera vaccine was an unheated agar emulsion killed by one per cent. phenol in which four strains were used in the manufacture, three of which were local, and the fourth Indian. Fifteen thousand doses were issued, the bacillary content being 2,000 million per cubic centimetre.

